# Supplementary material for: Empirical methods for controlling false positives and estimating confidence in ChIP-Seq peaks
Source: BMC Bioinformatics. 2008 Dec 5;9:523. doi: 10.1186/1471-2105-9-523 (PMC2628906; doi:10.1186/1471-2105-9-523)
Supplement: Additional File 1 — A variety of html documents from the USeq web site detailing the available applications, their best usage, output file type descriptions, command line menus, etc. [file 1471-2105-9-523-S1.zip › USeqUserGuides/JavaDoc.zip!/JavaDoc/trans/graphics/AggregatePlots.html]

AggregatePlots


|  |  |  |
| --- | --- | --- |
| **Overview** \| **Package** \| **Class** \| **Use** \| **Tree** \| **Deprecated** \| **Index** \| **Help** | |  |
| PREV CLASS   **NEXT CLASS** | **FRAMES**    **NO FRAMES**     **All Classes** |  |
| SUMMARY: NESTED \| FIELD \| CONSTR \| METHOD | DETAIL: FIELD \| CONSTR \| METHOD |  |


---


## trans.graphics Class AggregatePlots

```
java.lang.Object
  trans.graphics.AggregatePlots
```

---

``` public class AggregatePlots extends java.lang.Object ```

For each region finds the underlying scores from the chromosome specific bar files.
Converts the bps to zero coordinates and centers all of the scores relative to the largest region.
Generates aggregate plots.

---

**Constructor Summary**

`AggregatePlots(java.lang.String[] args)`


| **Method Summary** | |
| --- | --- |
| `void` | `loadChromosomeBarFile()` |
| `static void` | `loadRegions(Sgr sgr, Positive[] pos)`             Checks to see if an sgr object is in the positives, if so, adds the sgr object to the positive. |
| `static void` | `main(java.lang.String[] args)` |
| `static int` | `maxLength(Positive[] regions)`             Finds max length of the Region[], stop-start+1 |
| `void` | `plot()` |
| `static void` | `printArray(float[][] scores)`             Prints x as row, y as column with row index, float[y][x] |
| `static void` | `printDocs()` |
| `static void` | `printEachArray(float[][] scores)`             Prints x as row, y as column with row index, float[y][x] |
| `void` | `processArgs(java.lang.String[] args)`             This method will process each argument and assign new varibles |

**Methods inherited from class java.lang.Object**

`equals, getClass, hashCode, notify, notifyAll, toString, wait, wait, wait`

**Constructor Detail**

### AggregatePlots

```
public AggregatePlots(java.lang.String[] args)
```


**Method Detail**

### plot

```
public void plot()
```

---


### printArray

```
public static void printArray(float[][] scores)
```

:   Prints x as row, y as column with row index, float[y][x]

---


### printEachArray

```
public static void printEachArray(float[][] scores)
```

:   Prints x as row, y as column with row index, float[y][x]

---


### maxLength

```
public static int maxLength(Positive[] regions)
```

:   Finds max length of the Region[], stop-start+1

---


### loadChromosomeBarFile

```
public void loadChromosomeBarFile()
```

---


### loadRegions

```
public static void loadRegions(Sgr sgr,
                               Positive[] pos)
```

:   Checks to see if an sgr object is in the positives, if so, adds the sgr object to the positive.

---


### main

```
public static void main(java.lang.String[] args)
```

---


### processArgs

```
public void processArgs(java.lang.String[] args)
```

:   This method will process each argument and assign new varibles

---


### printDocs

```
public static void printDocs()
```


---


|  |  |  |
| --- | --- | --- |
| **Overview** \| **Package** \| **Class** \| **Use** \| **Tree** \| **Deprecated** \| **Index** \| **Help** | |  |
| PREV CLASS   **NEXT CLASS** | **FRAMES**    **NO FRAMES**     **All Classes** |  |
| SUMMARY: NESTED \| FIELD \| CONSTR \| METHOD | DETAIL: FIELD \| CONSTR \| METHOD |  |


---
